# lncRNA CRNDE promotes the proliferation and metastasis by acting as sponge miR-539-5p to regulate POU2F1 expression in HCC

**DOI:** 10.1186/s12885-020-06771-y

**Published:** 2020-04-06

**Authors:** Zhixi Li, Gang Wu, Jie Li, Youyu Wang, Xueming Ju, Wenjun Jiang

**Affiliations:** 1 Department of Pediatric Surgery, Sichuan Academy of Medical Sciences and Sichuan Provincial People’s Hospital, Chengdu, 610072 China; 2 Department of Hepatobiliary Surgery, Sichuan Academy of Medical Sciences and Sichuan Provincial People’s Hospital, Chengdu, 610072 China; 3 Department of Ophthalmology, Sichuan Academy of Medical Sciences and Sichuan Provincial People’s Hospital, Chengdu, 610072 China; 4 Department of Thracic Surgery, Sichuan Academy of Medical Sciences and Sichuan Provincial People’s Hospital, Chengdu, 610072 China; 5 Department of Ultrsound, Sichuan Academy of Medical Sciences and Sichuan Provincial People’s Hospital, Chengdu, 610072 China

**Keywords:** HCC, lncRNA CRNDE, miR-539-5p, POU2F1, ceRNA

## Abstract

**Background:**

This article focuses on the roles and mechanism of lncRNA CRNDE on the progression of HCC.

**Methods:**

We used qRT-PCR to detect the expression of lncRNA CRNDE in HCC cells, normal cells and clinical tissues. MTT assay, FCM analysis, Transwell migration and invasion assay were used to detect the effects of lncRNA CRNDE on cell viability, apoptosis, migration and invasion of HCC cells. The expression of apoptosis-related proteins Bcl-2, Bax, Cleaved Caspase 3, Cleaved Caspase 9, EMT epithelial marker E-cadherin and mesothelial marker Vimentin were analyzed by Western blot. Online prediction software was used to predict the binding sites between lncRNA CRNDE and miR-539-5p, or miR-539-5p and POU2F1 3’UTR. Dual luciferase reporter assay, qRT-PCR and RNA pulldown were used to detect target-relationship between lncRNA CRNDE and miR-539-5p. Dual luciferase reporter assay, qRT-PCR, Western blot and Immunofluorescence were used to detect target-relationship between miR-539-5p and POU2F1. qRT-PCR was used to detect the expression of miR-539-5p and POU2F1 in clinical tissues. Rescue experiments was used to evaluate the association among lncRNA CRNDE, miR-539-5p and POU2F1. Finally, we used Western blot to detect the effects of lncRNA CRNDE, miR-539-5p and POU2F1 on NF-κB and AKT pathway.

**Results:**

lncRNA CRNDE was highly expressed in HCC cells and HCC tissues compared with normal cells and the corresponding adjacent normal tissues. lncRNA CRNDE promoted the cell viability, migration and invasion of HCC cells, while inhibited the apoptosis and promoted the EMT process of HCC cells. lncRNA CRNDE adsorbed miR-539-5p acts as a competitive endogenous RNA to regulate POU2F1 expression indirectly. In HCC clinical tissues, miR-539-5p expression decreased and POU2F1 increased compared with the corresponding adjacent normal tissues. lncRNA CRNDE/miR-539-5p/POU2-F1 participated the NF-κB and AKT pathway in HCC.

**Conclusion:**

lncRNA CRNDE promotes the expression of POU2F1 by adsorbing miR-539-5p, thus promoting the progression of HCC.

## Background

Hepatocellular carcinoma (HCC) which has a poor prognosis and high mortality rate, is one of the most malignant cancer worldwide. About 750,000 new cases and 700,000 death cases occur every year in the world [1, 2]. HCC ranks as the third leading cause of cancer-related death worldwide [3]. HCC lacks typical clinical symptom in the early stage and is difficult to diagnose early. Once the typical symptom appears, the tumor is in the advanced stage with a poor prognosis and low 5-year survival rate [4]. Thus, a deep understanding of the pathogenesis and molecular mechanism are contribute to the diagnosis and treatment of patients with HCC.

The ENCODE program has clarified that approximately 90% of human genomic DNA sequences are capable of being actively transcribed, whereas only 2% of these transcripts are capable of encoding proteins and the remaining large transcripts are referred to as non-coding RNAs (ncRNAs). Long non-coding RNAs (lncRNAs) and microRNAs (miRNAs) are the major components of non-coding RNA [5–8]. LncRNA is a group of more than 200 nucleotides in length and has no protein-coding function. A large number of studies have shown that lncRNA plays important roles in many biological processes, including cell proliferation, migration [9], differentiation [10] and apoptosis [11]. Current researches suggest that the underlying mechanisms of lncRNAs is diverse, including regulation of chromatin remodeling, histone modifications and as competitive endogenous RNAs [12, 13]. However, the current research on the roles of lncRNA in HCC is still limited, and the understanding of the molecular mechanism is not clear.

lncRNA CRNDE (Colorectal Neoplasia Differentially Expressed) is located on human chromosome 16, which is highly expressed in various of cancer and acts as a cancer-promoting function. For example, lncRNA CRNDE is an important serological marker in the diagnosis and prognosis of colon cancer [14, 15]. In breast cancer, lncRNA CRNDE plays an important role by activating Wnt/β-catenin signal pathway and adsorbing miRNA-136 [16]. lncRNA CRNDE is involved in the radiotherapy tolerance of lung cancer by regulating the expression of p21 in lung cancer [17]. However, the roles and mechanism of lncRNA CRNDE specifically involved in the process of HCC is still unclear.

miRNA is a class of non-coding RNA of approximately 22 nt that regulates gene expression at the post-transcriptional level by target mRNA 3’UTR [18]. Increasing studies have shown that miRNAs play important roles in the malignant process of HCC. For example, miRNA-17-5p inhibits proliferation and initiates apoptosis by targeting TGFβR2 in lung cancer [19]; MiR-424 targets the oncogene TNFAIP1 and promotes metastasis in lung cancer [20, 21]. In this study, we selected miR-539-5p which targets lncRNA CRNDE for further research. Studies have shown that miR-539-5p is involved in the malignant progression of nasopharyngeal carcinoma cells [22]. However, the roles of miR-539-5p in HCC has not been reported. In addition, we interpret the molecular mechanism by searching for its target gene POU2F1. POU2F1 is also an important and widely studied factor that plays an important role as a cancer-promoting factor in cervical cancer [23, 24] and osteosarcoma [25–27].

This study mainly explored the effects and mechanism of lncRNA CRNDE on the proliferation, migration and invasion of HCC. It is believed that the interaction network of 1ncRNA miRNA and mRNA can better understand the pathogenesis of HCC at a molecular level from a new perspective.

## Methods

### Cells and clinical tissues

Experiments were performed using one normal hepatocyte (HL-7702), three hepatocellular carcinoma cell lines (HepG2, Huh-7 and QGY-7703). All cell lines were purchased from the American Type Culture Collection (ATCC) cell bank and were frozen and recovered in Key Laboratory of Sichuan Academy of Medical Sciences & Sichuan Province People’s Hospital (Chengdu, China) from Augst 2017. No mycoplasma contamination was existed in the cultured cells. Cell lines used in this article was required ethics approval. HL-7702, HepG2 and Huh-7 were cultured in DMEM and QGY-7703 was cultured in RPMI-1640 containing 10% fetal calf serum 100 μg/mL penicillin and 100 units/mL streptomycin at 37 °C, 5% CO_2_. Primary tumor tissue and paired non-tumor tissue samples were obtained from Sichuan Academy of Medical Sciences & Sichuan Provincial People’s Hospital. Twenty patients with HCC were invited to participated in this research. All patients had not received treatment before surgery. Written informed consent has been obtained from each subject and the research method has been approved by the ethics committee of Sichuan Academy of Medical Sciences & Sichuan Provincial People’s Hospital.

### Plasmid construction and cell transfection

The full-length of human lncRNA CRNDE and POU2F1 was synthesized by Invitrogen (Shanghai, China) and cloned into the vector pmirGLO or pCDNA3 (Clontech Laboratories, Inc., San Francisco, CA). miR-539-5p mimics, inhibitor and negative control (NC) were synthesized by GenePharma (Shanghai, China). The cells were seeded in 6-well plates overnight (1 × 10^6^/well), and then lncRNA (GenePharma), miRNA or plasmid were transfected with liposome Lipofectamine 2000 (Invitrogen) according to the instructions. The cells were changed to normal culture solution 6 h after transfection, 48 h and then proceed to the next test.

### Cell viability

Cell viability was measured by MTT assay. Briefly, after transfection of cells 48 h, 96-well plates were seeded at a cell density of 4 × 10^3^ cells/well, transfected at a density of 60–70% after adherence, and 20 μl MTT (5 mg/ml; Sigma-Aldrich) was added at 24 h, 48 h, and 72 h after transfection. After incubating for 4–6 h in a 37 °C incubator of % CO_2_, the culture solution was discarded, 100 μl of DMSO was added for 10 min, and OD450 was detected by a microplate reader. Three independent replicate experiments were performed.

### qRT-PCR

QRT-PCR detection of cells 48 h after transfection. Total RNA extraction, RNA reverse transcription and qRT-PCR reaction were performed according to the kit instructions. The qRT-PCR reaction system was thoroughly mixed in a 96-well plate, centrifuged at 3000 r/min for 3 min, and then subjected to fluorescence quantitative PCR instrument for reaction. Each sample and the detected gene were set to 3 replicates. The qRT-PCR reaction was carried out with ACTB as the internal reference gene. The reaction conditions were: pre-denaturation at 95 °C for 3 min, denaturation at 95 °C for 2 s, annealing at 60 °C for 20 s, and extension at 72 °C for 1 min for a total of 40 cycles. The ratio of the expression of the target molecule in the experimental group and the control group was expressed as a multiple = 2-^ΔΔCt^. Each experiment was repeated 3 times independently.

### Western blot

Forty-eight h after transfection of cells, trypsinize and collect the cells of each group, centrifuge at 700 r/min for 5 min, resuspend the cells in PBS for 2 times, add the cell lysate RIPA, thoroughly blow the cell pellet, and lyse on ice bath for 30 min. After centrifugation at 12000 r/min for 10 min, the supernatant was collected, and an appropriate amount of protein loading buffer was added in proportion, and boiled for 10 min to obtain a total protein sample of the cells. A 10% concentration of sodium dodecyl sulfate polyacrylamide gel (SDS-PAGE) was prepared for loading, electrophoresis, and electroporation. After the electroporation, the PVDF membrane containing the target protein was placed in a TBST-containing 5% skim milk containing gel. In the solution, the cells were blocked at room temperature for 2 h, the primary antibody Bax(1:1000), Bcl-2(1:1000), Cleaved Caspase 3 (1:500), Caspase 3(1:500), E-cadherin(1:1000), Vimentin(1:1000), POU2F1(1:1000), p-IKB(1:500), IKB(1:1000), p-AKT(1:500), AKT(1:1000), p-ERK(1:300), ERK(1:1000), NF-KB(1:1000), GAPDH(1:2000) were incubated at 4 °C overnight, the TBST was eluted 3 times for 5 min, the second antibody was incubated for 2 h at room temperature, and then eluted 3 times with TBST for 5 min each time, and finally developed.

### Transwell migration and invasion assay

After transfection of cells 48 h, the cells were inoculated into a 24-well plate. 8 × 10^4^ cells were plated in each chamber. In the 24-well plate, medium without serum was added and 20% serum was added into each chamber. The Transwell chamber was taken out, washed once and fixed in anhydrous methanol for 20 min; After the fixation, the chamber was stained with 0.5% methyl violet dye solution for 15 min. Five different fields of view were randomly selected, photographed, and the number of cells migrated according to the records were counted, and finally statistical analysis was performed.

### FCM

After transfection of cells 48 h, frozen sections of liver tissue (5 μm) were subjected to FCM staining using a commercially available kit (Beyotime, China).

### Immunofluorescence

After transfection of cells 48 h, the cells climbed on the slides were washed with PBS. Paraformaldehyde fixed cells and PBS washed. 0.2% Trinton X incubated cell. After wash with PBS, cell was incubated with POU2F1 antibody 4 °C overnight. Next day, cell was washed with 0.2% Trinton X-100 PBS wash and incubated with secondary antibody in room temperature dark. Slide was covered with tablet containing DAPI and photographed by fluorescence microscopy.

### Luciferase reporter assay

We cloned the lncRNA CRNDE fragment containing the miR-539-5p target site or mutation site by PCR and then cloned into pmirGlO Dual-luciferase Vector (Promega) to construct the reporter vectors, lncRNA CRNDE Wt and lncRNA CRNDE Mut. The POU2F1 fragment containing the miR-539-5p target site or mutation site was amplified by PCR and cloned into pmirGlO Dual-luciferase miRNA Target Expression Vector (Promega) to construct the reporter vector POU2F1 3’UTR Wt and POU2F1 3 ‘UTR Mut. Cells were subjected to miR-539-5p mimic/inhibitors and lncRNA CRNDE wild type/mutant cotransformation or miR-539-5p mimic/inhibitors and POU2F1 Wt or Mut using Lipofectamine 2000 (Invitrogen, Carlsbad, CA) according to the manufacturer’s instruction. Fluorescence activity was measured using a dual luciferase assay as described after 48 h.

### RNA pulldown

lncRNA CRNDE sequence was labelled in Biotin and performed RNA pulldown using Pierce Magnetic RNA-Protein Pull-Down Kit (Thermo Fisher, USA). qRT-PCR assay was used to detect lncRNA CRNDE enrichment in the RNA fraction.

### Subcutaneous xenografts in nude mice

The cells were seeded in T75 culture bottle and transfected with liposome Lipofectamine 2000 (Invitrogen) according to the instructions. After transfection, G418 was used the screen the cells which stably exoressed lncRNA CRNDE. Nude mice was usd to purchased from Beijing Vital River Laboratory Animal Technology Co., Ltd. Stably expressed cells (2 × 10^6^ cells per mice) was injected into subcutaneous of right scapula. Four weeks later, tissues was taken off, measured tumor size and weight.

### Statistical analysis

All analyses were carried out using SPSS 18.0 software (SPSS Inc., Chicago, IL, USA). Paired Student’s t-test, Mann-Whitney U test, and one-way analysis of variance were used in the study to evaluate statistical differences. *p* < 0.05 was considered statistically significant.

## Results

### lncRNA CRNDE was upregulated in HCC cells and clinical tissues

**Fig. 1:**
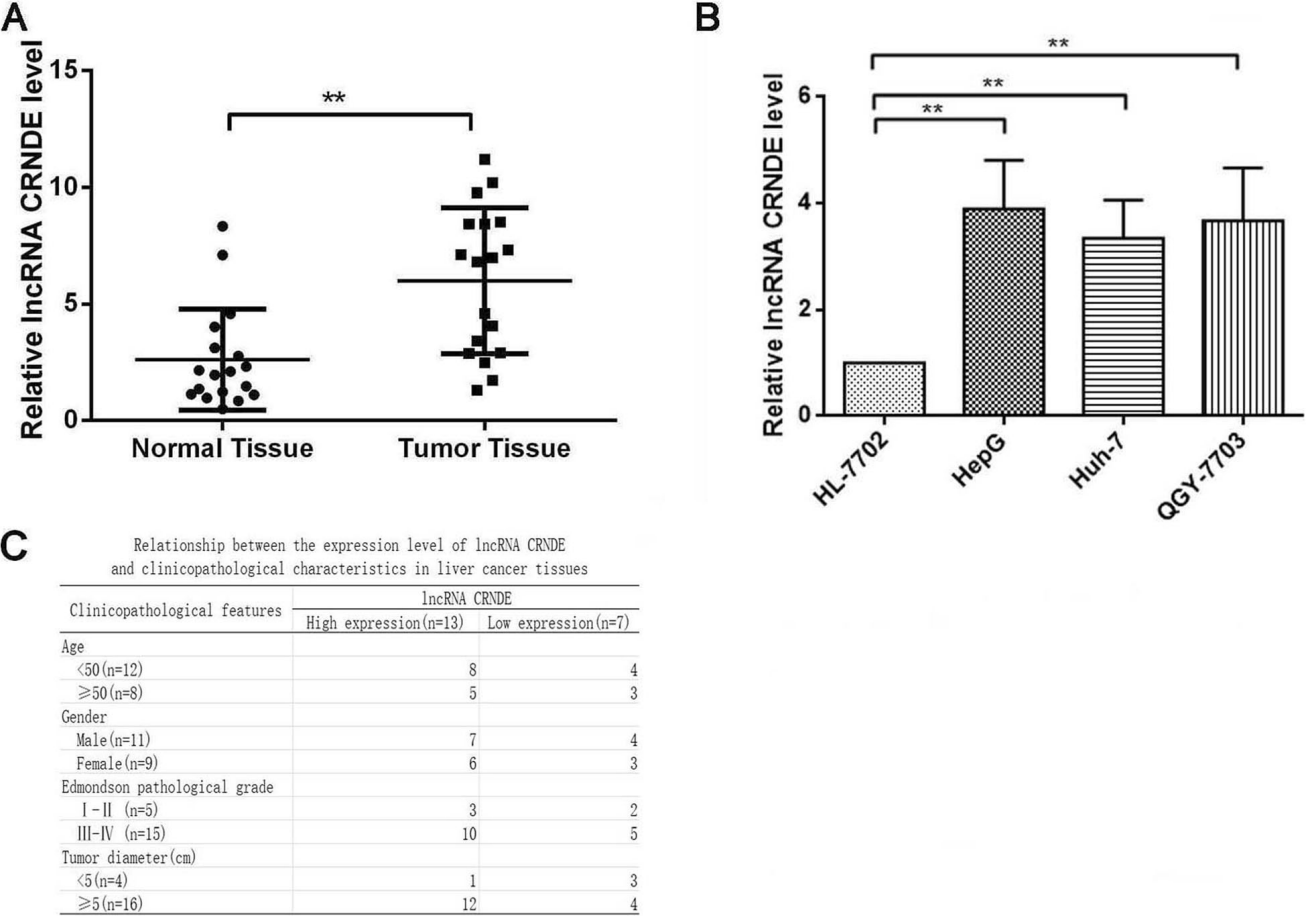
lncRNA CRNDE expression was upregulated in HCC cells and clinical tissues. **a** lncRNA CRNDE relative expression levels were determined in 18 paired HCC clinical tissues and their corresponding normal samples. The level of lncRNA CRNDE expression was normalized to GAPDH. **b** qRT-PCR analysis of lncRNA CRNDE expression in three HCC cells and one cultured human liver epithelial cells. **c** Correlation analysis was used to detect the expression of lncRNA CRNDE in HCC and clinicopathological indicators. All bars indicate the means±SD. All experiments were performed independently at least three times

To indentify the roles of lncRNA CRNDE on the development of HCC, we detected the expression level of lncRNA CRNDE in HCC clinical tissues and cells. qRT-PCR showed that compared with the adjacent normal tissues, lncRNA CRNDE was highly expressed in HCC tissues (Fig. 1a). Meanwhile, qRT-PCR showed that compared with the normal hepatocytes cells (HL-7702), lncRNA CRNDE was highly expressed in HCC cells (HepG2, Huh-7 and QGY-7703) (Fig. 1b). Subsequently, we analyzed the correlation between the expression of lncRNA CRNDE in HCC and clinicopathological indicators (Fig. 1c). High expresssion of lncRNA CRNDE was associated with Edmondson pathological grade and tumor diameter positively.

### lncRNA CRNDE promoted proliferation, migration and invasion in HCC cells

**Fig. 2:**
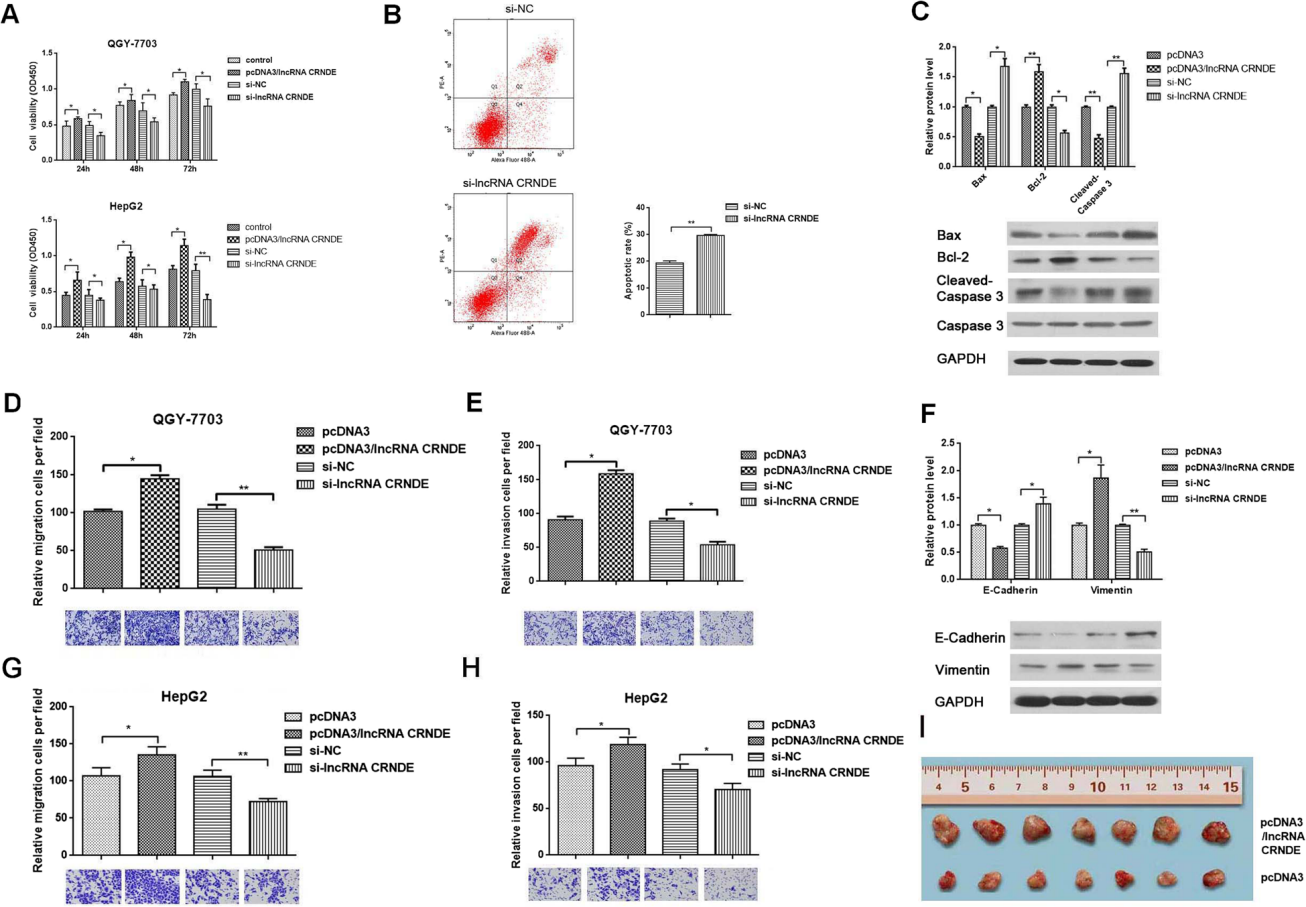
Knockdown of lncRNA CRNDE inhibits proliferation and metastasis in HCC cells. We divided the cells into four groups: pcDNA3, pcDNA3/lncRNA CRNDE, si-NC, and si-lncRNA CRNDE, transfected for 48 h, and then performed subsequent experiments. **a** MTT assay was performed to detect the proliferation ability of HCC cells when lncRNA CRNDE was overexpressed or knocked down. **b** FCM of apoptosis in HepG2 cells transfected with silncRNA CRNDE and siNC. **c** qRT-PCR and Western blot was used to detect the effects of lncRNA CRNDE on apoptosis associated genes. **d, e** Transwell migration and invasion assays were performed to detect the migration and invasion ability of QGY-7703 cells when lncRNA CRNDE was overexpressed or knocked down. **f** qRT-PCR and Western blot was used to detect the effects of lncRNA CRNDE on EMT associated genes. **g**, **h** Transwell migration and invasion assays were performed to detect the migration and invasion ability of HepG2 cells when lncRNA CRNDE was overexpressed or knocked down. **i** Tumor tissues in nude mice. *p < 0.05, **p < 0.01. All bars indicate the means±SD. All experiments were performed independently at least three times

To further explore the roles of lncRNA CRNDE in HCC, we constructed lncRNA CRNDE overexpression plasmid (pcDNA3/lncRNA CRNDE) and knockdown plasmid (si-lncRNA CRNDE). MTT assay was used to assess the effect of lncRNA CRNDE on cell viability in QGY-7703 and HepG2 cells. The results showed that overexpression of lncRNA CRNDE promoted cell viability, and knockdown of lncRNA CRNDE inhibited cell viability (Fig. 2a). FCM showed that cell apoptosis was promoted after knockdown of lncRNA CRNDE (Fig. 2b). Western blot revealed that overexpression of lncRNA CRNDE decreased the expression of Bax and Cleaved Caspase 3, and enhanced the expression of Bcl-2, while knockdown of lncRNA CRNDE increased the expression of Bax and Cleaved Caspase 3, and decreased the expression of Bcl-2(Fig. 2c). We also investigated the roles of lncRNA CRNDE on migration and invasion of HCC cells. As shown in Transwell assays, overexpression of lncRNA CRNDE accelerated cell migration and invasion in QGY-7703, whereas knockdown of lncRNA CRNDE attenuated its migration and invasion (Fig. 2d and e). Similar results were shown in HepG2 cells (Fig. 2g-h). We further performed Western blot to determine the biomarkers in the EMT process, which is a well-recognized migration and invasion mechanism in cancer. lncRNA CRNDE inhibited the expression of epithelial marker E-cadherin, and promoted the expression of Vimentin, classical mesenchymal markers, while knockdown of lncRNA CRNDE promoted the expression of E-cadherin and inhibited Vimentin expression (Fig. 2f). Finally, we screened stably expression cells which stably promoted lncRNA CRNDE expression in HepG2 cells and established subcutaneous xenografts model. As shown in Fig. 2i, lncRNA CRNDE promoted the tumor formation of HCC. These data indicated that lncRNA CRNDE could promote the proliferation, migration and invasion owing to inhibit apoptosis and promote EMT progression in HCC cells.

### lncRNA CRNDE acts as a ceRNA by sponging miR-539-5p and regulated POU2F1 expression indirectly

**Fig. 3:**
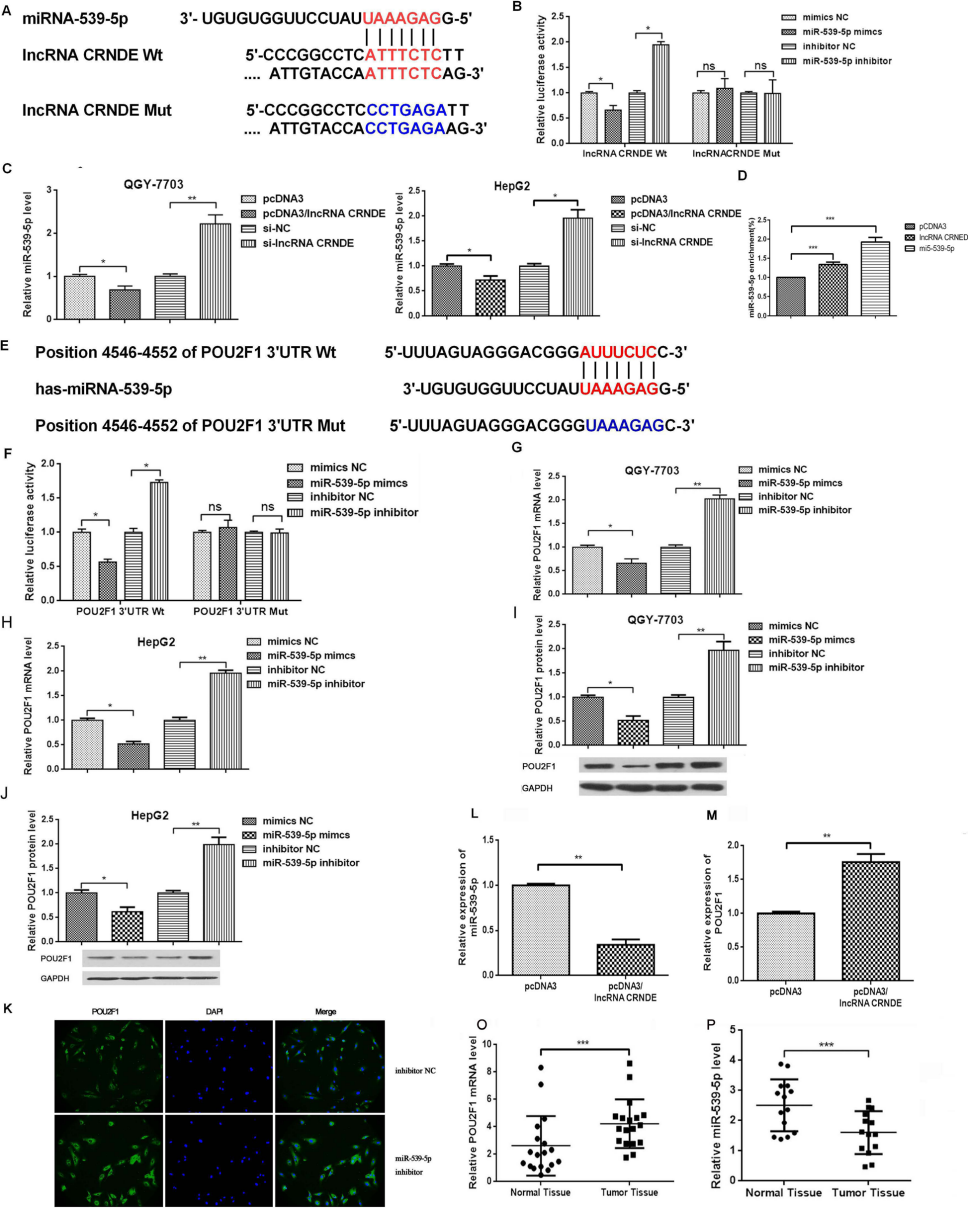
lncRNA CRNDE acted as a ceRNA by sponging miR-539-5p and regulated POU2F1 expression indirectly. We divided the cells into four groups: mimics NC, miR-539-5p mimics, inhibitor NC, and miR-539-5p inhibitor, transfected for 48 h, and then performed subsequent experiments. **a** The putative target sites between lncRNA CRNDE and miR-539-5p were shown. **b** Dual-luciferase analysis was proformed when HCC cells were co-transfected with lncRNA CRNDE-wt and miR-539-5p mimic or lncRNA CRNDE-mut and miR-539-5p mimic. The activity of luciferase was detected. **c** miR-539-5p expression level was detected by qRT-PCR when lncRNA CRNDE was overexpressed or knocked down. **d** RNA pulldown was used to presented the binding between lncRNA CRNDE and miR-539-5p as fold enrichment. **e** Prediction program was used to find that there is a putative target of miR-539-5p in the POU2F1 sequences. **f** Dual-luciferase analysis showed that effects of miR-539-5p on the luciferase activity of constructs of the type binding site. qRT-PCR (**g**) and Western blot (**j**) were used to detect the POU2F1 level when the miR-539-5p was overexpressed or knocked down. miR-539-5p. **k** Immunofluorescence was used to detect the expression of POU2F1 in HepG2 cells. qRT-PCR (**l**) and Western blot (**m**) were used to detect the level of miR-539-5p and POU2F1 in nude tissues. **o** and POU2F1 (**p**) relative expression levels were determined in 18 paired HCC clinical tissues and their corresponding normal samples.*p < 0.05, **p < 0.01. All bars indicate the means±SD. All experiments were performed independently at least three times

Current studies showed that lncRNA could act as ceRNA to regulate mRNA expression through matching miRNAs. An analysis of online prediction software MicroInspector database revealed that lncRNA CRNDE harbors miR-539-5p binding site (Fig. 3a). To validate the targeting relationship between lncRNA CRNDE and miR-539-5p, we constructed the pmirGLO/lncRNA CRNDE plasmid by inserting lncRNA CRNDE sequence into pmirGLO luciferase reporter plasmid. As shown in Fig. 3b, co-transfected wild-type pmirGLO/lncRNA CRNDE with miR-539-5p decreased the luciferase activity, and when mutated the binding sites in pmirGLO/lncRNA CRNDE-Mut, no significant change was observed, indicating the sequence-specific binding of miR-539-5p to lncRNA CRNDE. qRT-PCR verified that overexpression of lncRNA CRNDE decreased the level of free miR-539-5p, and knockdown of lncRNA CRNDE increased the level of free miR-539-5p in QGY-7703 and HepG2 cells (Fig. 3c). Consistently, RNA pulldown revealed that lncRNA CRNDE could target miR-539-5p directly (Fig. 3d).

Many previous studies showed that miRNAs play biological functions by targeting mRNA 3’UTR. POU2F1 was predicted to act as the downstream target of miR-539-5p through complementary binding sequence in the POU2F1 3’UTR (Fig. 3e). The predicted and mutanted binding sites in the 3’UTR were constructed into the pmirGLO luciferase reporter plasmid. As shown in Fig. 3f, when co-transfection with pmirGLO/POU2F1 3’UTR wide type reporter, miR-539-5p decreased and knockdown miR-539-5p elevated fluorescence activity. When co-transfection with pmirGLO/POU2F1 3’UTR mutation, no significant changes of fluorescence activity were observed. qRT-PCR and Western blot showed a significant inhibition of POU2F1 mRNA and protein level after overexpression of miR-539-5p in QGY-7703 and HepG2 cells (Fig. 3g-j). Immunofluorescence showed that miR-539-5p inhibitor increased the expression of POU2F1 in HepG2 cells (Fig. 3k). In vivo, we found that the expression of miR-539-5p inhibited and POU2F1 promoted in the tissues in pcDNA3/lncRNA CRNDE group compared with pcDNA3 group (Fig. 3l, m). In clinical tissues, miR-539-5p expression decreased and POU2F1 increased in HCC clinial tissues compared with the corresponding adjacent normal tissues (Fig. 3o, p). These results indicated that lncRNA CRNDE acts as an endogenous sponge by binding miR-539-5p and indirectly regulates POU2F1 expression.

### The promoting effects of lncRNA CRNDE could be reversed by miR-539-5p and POU2F1

**Fig. 4:**
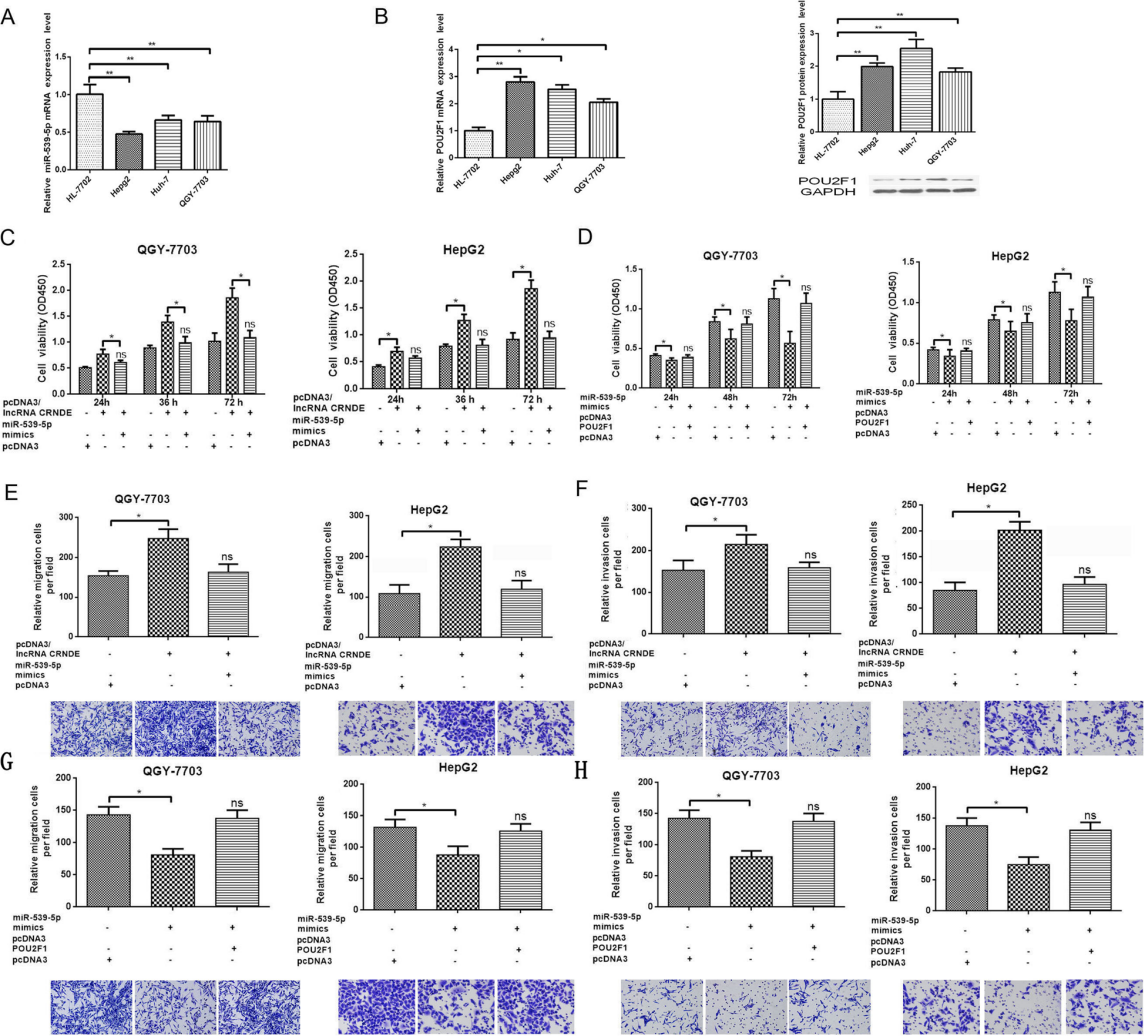
The promoting effects of lncRNA CRNDE can be reversed by miR-539-5p and POU2F1. **a** qRT-PCR analysis of miR-539-5p expression in three HCC cells and one cultured human liver epithelial cells. **b** qRT-PCR analysis of POU2F1 expression in three HCC cells and one cultured human liver epithelial cells. Western Blot analysis of POU2F1 expression in three HCC cells and one cultured human liver epithelial cells. **c** We divided the cells into groups: pcDNA3/lncRNA CRNDE, miR-539-5p mimics, pcDNA3, transfected for 48 h. MTT assays were performed to detect the proliferation ability of QGY-7703 and HepG2 cells. **d** We divided the cells into groups: miR-539-5p mimics, pcDNA3/POU2F1, pcDNA3, transfected for 48 h. MTT assays were performed to detect the proliferation ability of QGY-7703 and HepG2 cells. **e, f** We divided the cells into three groups: pcDNA3/lncRNA CRNDE, miR-539-5p mimics, pcDNA3, transfected for 48 h. Transwell migration and invasion assays were performed to detect the migration and invasion ability of QGY-7703 and HepG2 cells. *p < 0.05, **p < 0.01. All bars indicate the means±SD. All experiments were performed independently at least three times. **g**, **h** We divided the cells into groups: miR-539-5p mimics, pcDNA3/POU2F1, pcDNA3, transfected for 48 h. Transwell migration and invasion assays were performed to detect the migration and invasion ability of QGY-7703 and HepG2 cells. *p < 0.05, **p < 0.01. All bars indicate the means±SD. All experiments were performed independently at least three times

To evaluate the association among lncRNA CRNDE, miR-539-5p and POU2F1, we designed rescue experiments and construsted POU2F1 overexpression plasimd (pcDNA3/POU2F1). As shown in Fig. 4a, overexpression of lncRNA CRNDE promoted cell viability, which could be abrogated by upregulation of miR-539-5p in QGY-7703 and HepG2 cells. Overexpression of miR-539-5p inhibited cell viability, which could be abrogated by upregulation of miR-539-5p in QGY-7703 and HepG2 cells (Fig. 4b). Transwell assays showed that overexpression of miR-539-5p could abrogated the promotion of cell migration and invasion after overexpression of lncRNA CRNDE in QGY-7703 and HepG2 cells (Fig. 4c, d). Overexpression of POU2F1 could abrogated the inhibition of cell migration and invasion made by overexpressing of miR-539-5p in QGY-7703 and HepG2 cells (Fig. 4e, f).

### lncRNA CRNDE/miR-539-5p/POU2F1 involved in NF-κB and AKT pathway

**Fig. 5:**
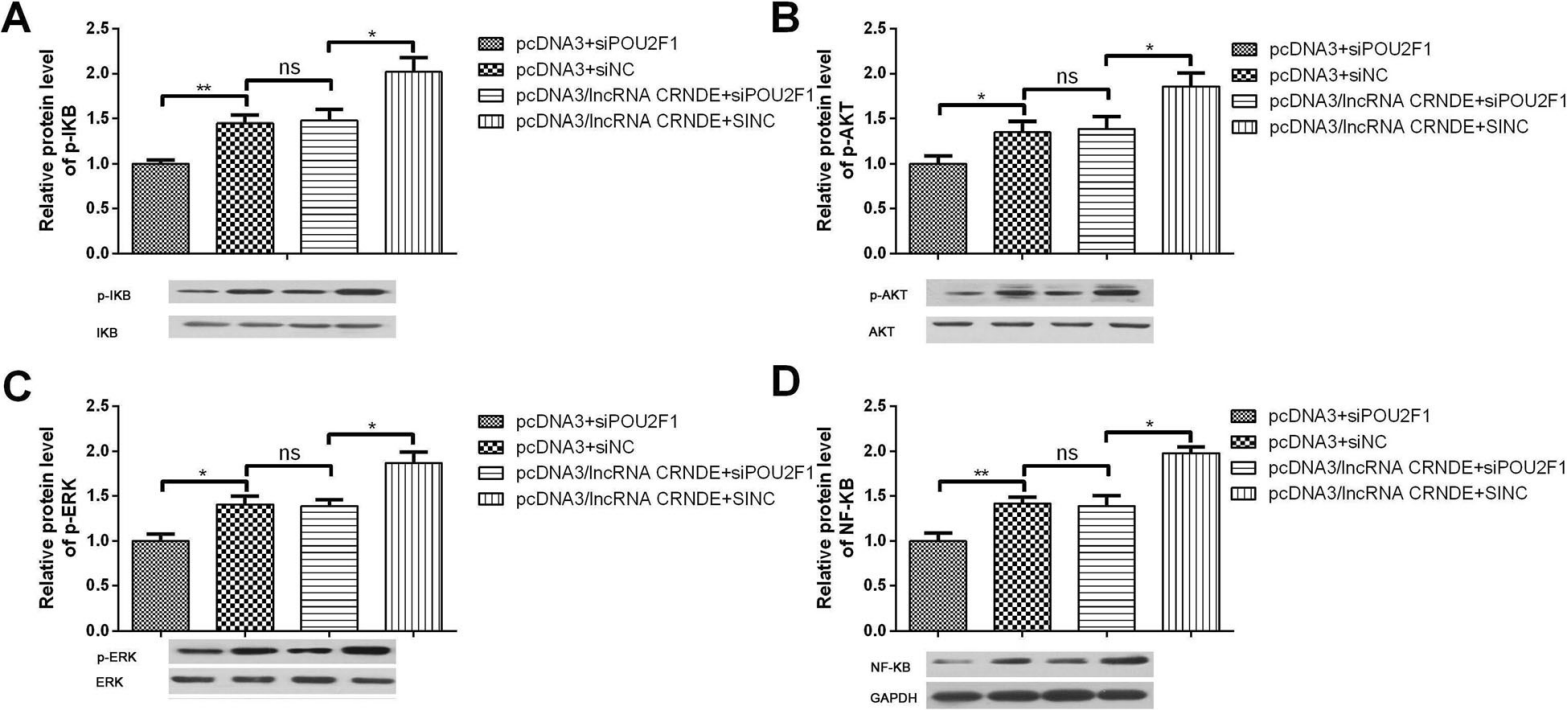
lncRNA CRNDE/miR-539-5p/POU2F1 involved in NF-κB and AKT pathway activity. We divided the cells into groups: pcDNA3 + siPOU2F1, pcDNA3 + siNC, pcDNA3/ lncRNA CRNDE+siPOU2F1, lncRNA CRNDE+siNC, transfected for 48 h. Western blot was performed to detect the effects of lncRNA CRNDE/miR-539-5p/POU2F1 on IKBa(**a**), AKT(**b**), ERK(**c**) phosphorylation level. **d** The NF-κB(p65) level in nuclear was detected when cells were treated with lncRNA CRNDE/miR-539-5p/POU2F1. *p < 0.05, **p < 0.01. All bars indicate the means±SD. All experiments were performed independently at least three times

In order to investigate whether NF-κB and AKT pathway were activated by the lncRNA CRNDE/miR-539-5p/POU2F1 axis in HepG2 cells, we estimated the phosphorylation level of IKB, AKT and ERK in the nucleus. The results showed that lncRNA CRNDE promoted the phosphorylation of IKB, AKT and ERK, while knockdown of POU2F1 synergistically abrogated the promotion of phosphorylation (Fig. 5a-c). Subsequently, we detected the effects of lncRNA CRNDE/miR-539-5p/POU2F1 axis on the level of NF-κB in the nucleus. As shown in Fig. 5d, lncRNA CRNDE promoted the level of NF-κB in the nucleus, while knockdown of POU2F1 synergistically abrogated the promotion of phosphorylation.

### The model of lncRNA CRNDE/miR-539-5p/POU2F1 in HCC

**Fig. 6:**
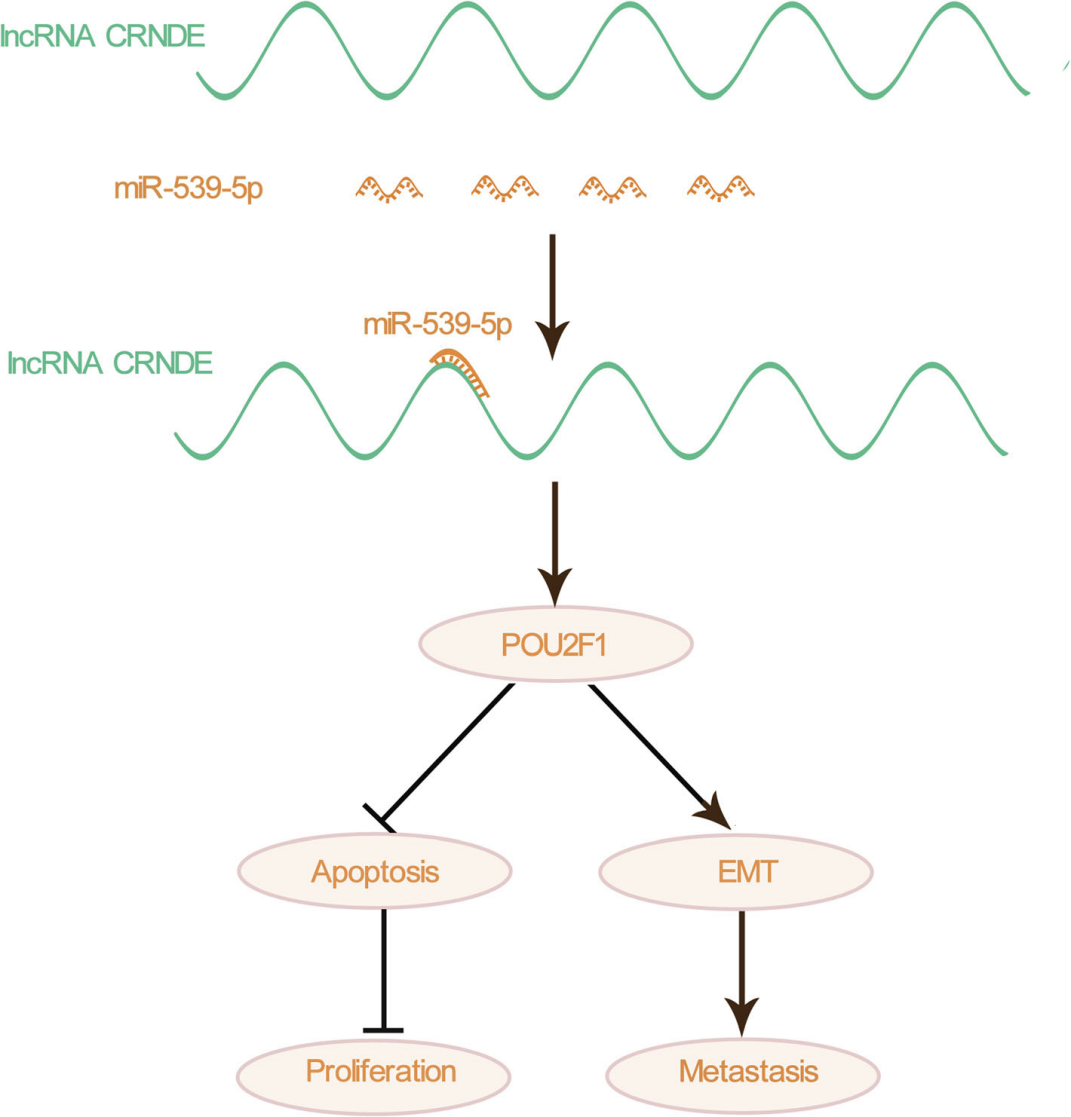
The model of lncRNA CRNDE/miR-539-5p/POU2F1 in HCC. The overview of this study

We summarized the interaction of lncRNA CRNDE, miR-539-5p, and POU2F1 in HCC. lncRNA CRNDE promotes proliferation by inhibiting apoptosis and promotes metastasis by regulating cellular EMT processes in HCC (Fig. 6).

## Discussion

Over the past decades, the discovery of lncRNAs has dramatically changed our biological understanding of many complex diseases. HCC is a research hot-spot and its prevention and treatment have been plaguing the researchers. Therefore, exploring the molecular biological mechanism of HCC is of great significance. It has been pointed out in the literature that lncRNA plays an important role in HCC [28].

This study focused on the role of lncRNA CRNDE in HCC cells. We found that lncRNA CRNDE is highly expressed in HCC clinical tissues and cells compared with normal tissues and cells. MTT assay, Transwell migration and invasion confirmed that lncRNA CRNDE could promote cell proliferation, migration and invasion. we also confirmed that the promotion of proliferation, migration and invasion by lncRNA CRNDE was owing to the inhibition of apoptosis and promotion of EMT progress. In the mechanism and action of lncRNA CRNDE, we confirmed that lncRNA CRNDE could adsorb miR-539-5p and miR-539-5p could regulate POU2F1 expression. Then, in the validation of the regulatory relationship of biological functions, we found that miR-539-5p acts as a suppressor gene to inhibit the cell viability, migration and invasion of HCC cells, and can rescue the growth-promoting and metastatic effects induced by lncRNA CRNDE.

In addition, we also found that lncRNA CRNDE could activate NF-κB and AKT signal pathway. The activation of NF-κB and AKT signal pathway in tumors had been extensively studied. We found that miR-539-5p could inhibit the activation of NF-κB and AKT signal pathway induced by lncRNA CRNDE in HepG2 cells, while lncRNA CRNDE and POU2F1 can synergistically activate NF-κB and AKT signal pathway.

## Conclusions

In summary, this study found that lncRNA CRNDE promoted cell proliferation owing to the inhibition of apoptosis in HCC. lncRNA CRNDE promoted migration and invasion owing to the promotion of EMT process. lncRNA CRNDE/miR-539-5p/POU2F1 axis was existed in HCC and could activated NF-κB and AKT signal pathway.

## Data Availability

All authors can guarantee the authenticity and usability of the data and materials in the article. All the data used in this study can be found in the main paper. The datasets of WB was uploaded in the supplement material and other datasets will be available from the corresponding author on reasonable request.

## Abbreviations

lncRNA: Long non-coding RNA
CRNDE: Colorectal Neoplasia Differentially Expressed
HCC: Hepatocellular carcinoma
FCM: Flow Cytometry
EMT: Epithelial-Mesenchymal Transition
qRT-PCR: Quantitative reverse transcriptase PCR
WB: Western blot
